# Navigating the Shadows of Others’ Traumas: An In-Depth Examination of Secondary Traumatic Stress and Psychological Distress among Rescue Professionals

**DOI:** 10.3390/bs14010021

**Published:** 2023-12-27

**Authors:** Nazia Noureen, Seema Gul, Aneela Maqsood, Humaira Hakim, Arooj Yaswi

**Affiliations:** 1 Department of Psychology, Foundation University, Rawalpindi 44000, Punjab, Pakistan; nazia.ch88@gmail.com; 2 General Studies Department, College of Humanities and Sciences, Prince Sultan University, Riyadh 11586, Saudi Arabia; sgul@psu.edu.sa (S.G.); ayaswi@psu.edu.sa (A.Y.); 3 National Centre for Research on Suicide Prevention, Department of Behavioral Sciences, Fatima Jinnah Women University, Rawalpindi 44000, Punjab, Pakistan; 4 Department of Behavioral Sciences, Fatima Jinnah Women University, Rawalpindi 44000, Punjab, Pakistan; humairahakim76@gmail.com

**Keywords:** secondary traumatic stress, psychological distress, rescue workers, occupational stress

## Abstract

Rescue workers, who often find themselves on the frontlines of traumatic events, face an increased risk of experiencing secondary traumatic stress (STS) and psychological distress (PD). The nature of their work, alongside professional factors, can influence the way these psychological aspects manifest and their level of severity. This study aimed to explore the relationship between STS and PD in rescue workers. Additionally, it sought to understand how factors such as age, years of experience, duration of work, training received and direct exposure to trauma explain significant variations in reporting to STS. To conduct this research, a cross-sectional study design was implemented involving a sample of 80 rescue workers from the Punjab province in Pakistan. Data was collected over eight weeks using the secondary traumatic stress scale (STSS-17) and the general health questionnaire (GHQ-12) as primary assessment tools. Participants’ data was analyzed through Pearson correlation analysis, *t*-tests, and ANOVA. A strong positive correlation between STS and PD among rescue workers was established. Age significantly explains variation in scores, with older workers displaying reduced STS and PD symptoms. Rescue workers working for longer hours reported elevated STS compared to those working shorter shifts. Workers with more extended professional experience showcased lower STS, highlighting the potential resilience acquired over time. The study also underscored the importance of training duration: longer, well-structured training was associated with decreased STS and PD. Interestingly, longer exposure to trauma was found to be related to lower STS scores, albeit this warrants further investigation. This study underscores the intertwined nature of STS and PD among rescue workers and the various modulating factors. The study paves the way for more comprehensive research, expanding geographically and demographically.

## 1. Introduction

In an ever-evolving global landscape, characterized by multifaceted challenges, the invaluable role of rescue workers is becoming increasingly apparent during the COVID-19 pandemic, their role was accepted and appreciated throughout the world [1]. These individuals serve as the frontline defense against adversities, be it natural calamities, pandemics, or man-made disasters such as terrorism, civil unrest, and accidents [1]. While the significance of their work is undeniable, especially in traumatic or challenging situations, it is not without consequences [2]. Poulin et al. [3] aptly highlight that the very nature of giving and helping can lead to adverse psychological outcomes, primarily stress. This opinion is further endorsed by Rauvola et al. [4] who postulated that simply by being in a position of aiding or rescuing traumatized individuals, one could be susceptible to secondary traumatic stress (STS). 

STS, often paralleled with post-traumatic stress disorder (PTSD), emerges not from directly experiencing trauma but from witnessing its consequences or hearing about it [5,6]. Symptoms including but not limited to re-experiencing trauma, emotional numbing or avoidance behaviors, and hyperarousal define this condition. Rescue workers, due to their proximity to trauma victims and the scenes of incidents, are inherently vulnerable to STS, a phenomenon that may have deep implications on their psychological well-being and operational efficiency [7,8].

Beyond STS, rescue workers can also experience a variety of emotional and psychological challenges, encompassed under the term psychological distress (PD) [9,10]. Ranging from feelings of sadness and hopelessness to severe mental health conditions, PD offers a more extensive exploration into the emotional toll faced by these workers [11]. While STS provides a trauma-specific lens, PD allows for a more generalized understanding of their emotional state, encompassing a wider spectrum of symptoms not strictly limited to traumatic exposure [12].

Despite the inherent relevance of understanding STS and PD in rescue workers, the existing body of literature, especially in the context of Punjab Province, remains sparse. With its unique socio-political context, the Punjab Province in Pakistan stands as a significant ground for understanding these psychological challenges [2]. From partition riots in 1947 to the recent incidents of terrorism and civil unrest, the province has witnessed troubling events that have necessitated the consistent involvement of rescue workers [2,10]. This continuous exposure to traumatic events not only places these workers at physical risk but also makes them susceptible to several psychological stressors [7,8]. Given the province’s unique geopolitical and socio-cultural landscape, understanding these phenomena in this specific context becomes paramount. Such understanding holds promise for several reasons.

Understanding the psychological complexities that rescue workers face—especially in the context of the Punjab Province—is not merely an academic effort but a pressing necessity. The overall well-being of these rescue workers emerges as a paramount concern. While their physical challenges are obvious and immediately evident, the silent toll on their psychological well-being often remains hidden. Continuous and repeated exposure to trauma, without appropriate psychological support and intervention, can gradually corrode their mental stamina [7,11]. This weakening can manifest in various ways, such as burnout, reduced operational efficiency, and an increased susceptibility to mental health disorders. As Pink et al. [13] and Yasien et al. [9] appropriately point out, the mental resilience of rescue workers plays a crucial role in their overall effectiveness and longevity in their profession.

Furthermore, from an operational standpoint, the psychological health of these workers directly correlates with the efficiency and efficacy of rescue operations [14]. In high-pressure situations, where lives often hang in balance like regions with long-standing conflicts [15], the mental agility, resilience, and emotional stability of rescue workers can significantly determine the outcome of their efforts [16]. Ensuring their psychological well-being is not just about their personal health; it is about optimizing their performance and, by extension, improving the outcomes of their crucial operations [10].

However, the implications of understanding secondary traumatic stress (STS) and psychological distress (PD) in rescue workers extend beyond the immediate operational context. For policymakers, the insights derived from such understanding offer a robust foundation for formulating targeted interventions. Policymakers can harness this knowledge to ensure that brave individuals, who risk their lives for the safety of others, are holistically equipped to handle the challenges of their job, both physically and mentally [17]. 

Considering the above, this study aims to understand the relationship between STS and PD among rescue workers in Punjab Province, Pakistan. It seeks to explain how various factors like age, duration of working, exposure to trauma, and training duration are related to STS and PD and how they impacted the phenomenon of STS and PD, filling a significant gap in the existing literature.

## 2. Background

### 2.1. Secondary Traumatic Stress: Unmasking the Silent Shadow of Rescuer Trauma

Secondary traumatic stress (STS) has emerged as a pivotal area of interest in psychological research, particularly over the last three decades. Conceptualized by Figley (1995), STS is defined as “the natural consequent behaviors and emotions resulting from knowing about a traumatizing event experienced by a significant other—the stress resulting from helping or wanting to help a traumatized or suffering person” [18]. This essentially captures the essence of STS as a form of trauma that isn’t directly experienced but is a consequence of exposure to another’s trauma [18].

Many professionals, especially those in helping professions such as therapists, counselors, and social workers, often bear the brunt of STS without even realizing it [18]. They become the secondary recipients of trauma as they routinely engage with individuals who have experienced primary trauma [8]. Their consistent and prolonged exposure to traumatic narratives, distressing emotions, and agonizing experiences can make them vulnerable to STS [7].

STS in Rescue Workers: Rescue workers, given the nature of their job, consistently find themselves in environments replete with trauma. Whether it is attending to victims of natural disasters, accidents, acts of terrorism, or other emergencies, they are routinely exposed to deeply distressing scenes and narratives. This exposure, both direct and indirect, renders them highly susceptible to STS [19].

Ogińska-Bulik et al. [20] delved deep into the manifestation of STS in healthcare professionals working with trauma victims. While their primary focus was on medical professionals, the parallels with rescue workers are strikingly evident. Their study underscored the elevated levels of STS among professionals consistently exposed to the trauma of those they aim to help.

Another study by Kindermann et al. [5] specifically zeroed in on emergency call-takers and dispatchers, exploring the prevalence and intensity of STS among them. Their study highlighted that a significant proportion of emergency rescue workers, despite not exhibiting overt symptoms, showed underlying signs of STS. The study shed light on the often-overlooked psychological cost that these brave individuals pay in their line of duty [5].

Factors Influencing STS Among Rescue Workers: The susceptibility of rescue workers to secondary traumatic stress (STS) is not a uniform phenomenon. Various intrinsic and extrinsic factors play a pivotal role in determining the intensity and prevalence of STS among these brave individuals. A deeper dive into the literature reveals a complex interplay of several determinants. 

Age, often seen as a marker of both physical and emotional resilience, has been identified as a significant factor. Recent research by Dworkin et al. [21] suggests that younger rescue workers, possibly due to lesser experience or inadequate coping mechanisms, might experience higher levels of STS compared to their older counterparts. However, it is essential to note that older workers, because of prolonged exposure over the years, could exhibit accumulated STS. The tenure of professional engagement or length of professional experience in rescue operations emerges as a crucial determinant [22]. Novices, because of the sheer shock of initial exposures, might exhibit heightened STS symptoms. On the contrary, veterans, due to their extended exposure over the years, could develop a more nuanced form of STS, often masked by their apparent resilience [22].

Training, ideally, should serve as a buffer against STS. However, its impact is twofold. Well-structured training modules that emphasize psychological resilience can significantly reduce STS susceptibility [23]. But, as Robinson et al. [24] note, training that unduly emphasizes traumatic narratives without providing coping mechanisms can inadvertently induce STS, and proper training and intervention programs help nurses and medical professionals cope with STS. The duration of work is also linked with the frequency and intensity of exposure to traumatic events due to long working hours is also directly correlated with workload, and plays a pivotal role in STS manifestation. A study by Hinderer et al. [25] found that the number of hours worked per shift was associated with greater STS. Also, the detailed study of Yoder [26] found that heavy workload causes secondary traumatic stress among nurses from different hospital wards, i.e., emergency unit, oncology unit, or intensive care unit, especially without adequate intervals of rest, exhibited exacerbated STS symptoms.

Beyond the mere frequency of deployment, exposure to trauma is crucial. Witnessing highly distressing scenes, especially involving children or extreme violence for an extended period, can lead to profound STS symptoms. As highlighted by Armes et al. [27], exposure to trauma is directly affecting the STS of the individuals at work. Similarly, Adriaenssens et al. [28], highlighted that workers having more trauma caseload and engaged with many different traumatized patients and individuals appeared to have reported intensified STS symptoms. Certain professions, such as therapists, typically have a minimal chance of direct exposure to work-related trauma. On the other hand, professionals like paramedics and rescue workers operate at the heart of traumatic situations, often experiencing direct exposure. A meta-analysis by Cieslak et al. [29] underscores this distinction, suggesting that prolonged direct exposure to trauma is associated with heightened levels of secondary traumatic stress (STS) and psychological distress.

Understanding STS, particularly its manifestation among rescue workers, is of paramount importance. As the world grapples with increasing challenges, the role of rescue workers becomes even more pivotal. Ensuring their psychological well-being is not just an ethical imperative but also critical for the efficacy of rescue operations. Exploring STS, its nuances, and its manifestation with respect to different sub-groups of rescue workers provides a solid foundation for tailoring interventions, policies, and training programs, aiming for a holistic approach to their well-being.

### 2.2. The Silent Echo: Psychological Distress and Its Repercussions among Rescue Workers

Understanding Psychological Distress: Psychological distress (PD) is a broad term within the realm of mental health research, capturing a range of feelings and symptoms. These might span symptoms ranging from mild unease or sadness to severe conditions like anxiety and depression [12]. Traditionally, PD has been conceptualized as an emotional response to external factors, challenges, or specific life events. More recently, PD has been understood as not merely a reaction to external circumstances but also the byproduct of internal cognitive processes and perceptions [30].

This form of distress, while common across different populations, tends to manifest uniquely among specific professional groups. Among these, rescue workers emerge as a demographic of particular interest, given their continuous exposure to traumatic and high-pressure environments.

Psychological Distress among Rescue Workers: When considering rescue workers, their encounters with trauma, disaster scenes, and life-threatening situations are almost a daily routine. While the immediate physical risks of their profession are evident, the underlying psychological consequences, especially PD, often remain not immediately apparent but significant.

A study by Yasien et al. [9] emphasized the unique manifestation of PD among rescue workers. The research underscored how rescue workers, due to their recurrent exposures to traumatic events, often develop a form of chronic PD, which can persist even in the absence of immediate triggers. Another recent study by Pink et al. [13] revealed that although fire and rescue and police groups have psychological distress due to dealing with traumatized people, they have lower levels of PD as compared to other people and have higher resilience maybe due to frequent exposure to trauma and getting used to it.

This manifestation of PD is intricately linked with several factors, some of which overlap with the determinants of secondary traumatic stress (STS). Understanding PD, especially its intricate relation with factors like trauma exposure, professional duration, and personal resilience, is pivotal. The unique context of rescue workers makes this understanding even more critical. As efforts to support these brave individuals evolve, comprehensive insights into PD can pave the way for holistic interventions that cater to both their physical and psychological well-being.

## 3. Purpose and Hypotheses

### 3.1. Purpose

The primary aim of this research is to delve into the interrelations of secondary traumatic stress (STS) and psychological distress (PD) among rescue workers, specifically within the context of Punjab Province, Pakistan. By understanding these dynamics, the study is expected to not only enrich the academic literature in this domain but also provide actionable insights that can inform policy-making, training programs, and intervention strategies for the welfare of rescue workers in Pakistan. Given the unique socio-political backdrop of the Punjab Province, marked by a history of terrorism, civil unrest, and natural disasters, the study assumes added relevance. Exploring how variables such as age, work duration, training duration, experience, and trauma exposure explain differential reporting on STS and PD can provide a comprehensive understanding that has implications both at an individual and systemic level.

### 3.2. Development of Hypotheses

The development of the hypotheses is grounded in existing literature and the gaps identified in it. Studies have highlighted a positive correlation between STS and PD [31,32]. Given that both constructs originate from exposure to trauma, although in different capacities; it is imperative to examine how rescue workers with varying profiles may respond to STS and PD experiences. 

Research has indicated varying susceptibility to STS and PD based on age, with both younger and older rescue workers exhibiting unique symptoms [33]. According to Myerson et al. [33], distress levels were negatively correlated to age, like the degree of distress, decreased with age. Similarly, Hale et al. [34] also found that there are different manifestations of PD at different ages with more PD at lower ages.

Duration of professional engagement and work experience, be it short-term or long-term—less than and more than 3 years respectively—has been identified as a significant determinant of both STS and PD [22]. The quality and duration of training, especially concerning coping mechanisms and emotional resilience, plays a pivotal role in shaping the manifestation of STS and PD [23,24].

With this background, the following hypotheses are posited:

- **H1**: A positive correlation exists between secondary traumatic stress and psychological distress.
- **H2**: There is a significant difference in STS with different age groups.
- **H3**: There is a significant difference in STS and psychological distress with varying durations of work.
- **H4**: There is a significant difference in STS and psychological distress based on the length of work experience.
- **H5**: There is a significant difference in STS with varying durations of training.
- **H6**: STS and psychological distress vary with exposure to trauma.

## 4. Measures and Methods

### 4.1. Participant’s Information

The study focused on a specific group of rescue workers situated in the Punjab region of Pakistan. Using a non-probability purposive sampling method, 80 rescue workers were selected, all of whom were male because most male workers work in rescue departments. The age bracket for participants ranged from 20 to 45 years. Exploring the age distribution, 81% of these rescue workers fell within the age range of 22–35 years, while the remaining 18.8% were between the ages of 35–45.

In terms of inclusion criteria, the study considered rescue workers who were professionally affiliated with rescue operations and had a minimum of ten years of education. On the other side, the study excluded participants who were not professionally involved in rescue operations and those lacking formal education.

### 4.2. Measures Used for the Research

#### 4.2.1. Secondary Traumatic Stress Scale (STSS-17)

Bride and Figley in their foundational research discussed the development and validation of the STSS. This research validated STSS as an appropriate tool for assessing secondary traumatic stress, particularly due to its focus on key symptomatology areas such as intrusion, avoidance, and arousal [35]. Similarly, Jenkins and Baird’s study provides information about the application of STSS in different contexts, particularly in validating the concept of secondary traumatic stress and its measurement [36].

The secondary traumatic stress scale (STSS) is a 17-item instrument with Likert-type choices developed to measure intrusion, avoidance, and arousal symptoms associated with indirect exposure to traumatic events through an individual’s professional relationship with traumatized clients [35]. STSS scale has three subscales with Intrusion Subscale (items 2, 3, 6, 10, 13); the Avoidance Subscale (items 1, 5, 7, 9, 12, 14, 17); and the Arousal Subscale (items 4, 8, 11, 15, 16) [35]. Responses ranged from 1-5 in Likert form with (1 = never; 2 = rarely; 3 = occasionally; 4 = often; 5 = very often).

For a better understanding of participants, the secondary traumatic stress scale was translated into Urdu—a national language of Pakistan, and almost all people know the language. The translation procedure used for scale involved the contribution of 6 specialists (bilinguals) at various stages of translation. Firstly, the scales were given to three different individuals, who were skillful and talented in both English and Urdu. The individuals were required to translate the items into Urdu. These Urdu translations were then given to three other individuals who translated the items into English. These back translations were then compared to the original items and those Urdu statements whose English translations matched the original items were included and finally Urdu version of STSS was ready to administer.

#### 4.2.2. General Health Questionnaire (GHQ-12)

In the dynamic field of mental health research, the choice of appropriate tools for assessing psychological distress is pivotal. The general health questionnaire (GHQ-12), developed by Goldberg (1992), has stood out as a particularly effective instrument for this purpose. Its robust psychometric properties and multi-factor structure, explored by researchers like Gao et al. affirm its capability to comprehensively measure various facets of psychological distress [37]. The GHQ-12’s widespread application across different cultural and occupational settings, as reviewed by Jackson (2007), further testifies to its versatility and relevance [38]. This instrument’s efficacy is echoed in diverse studies, including Kim et al. (2013), which emphasize its utility in cross-cultural contexts [39]. Additionally, Ware et al.’s (1996) exploration into health survey construction offers insights into the reliability and validity of such tools, underlining the importance of nuanced, accurate assessments in mental health research [40].

The GHQ-12, developed as a short version of the general health questionnaire, is a self-report psychiatric screening tool used to detect psychiatric disorders in both clinical and non-psychiatric settings [41]. 

This questionnaire, originally a 60-item instrument introduced by David Goldberg in 1978, was shortened to a 12-item version in 1988 [42]. GHQ-12 evaluates various domains like depression, anxiety, social disturbance, and hypochondriasis, focusing on the severity of a mental problem experienced in the preceding weeks. Using a 4-point Likert scale (ranging from 0–3), the GHQ-12 yields a total score between 0 and 36, where a higher score indicates pronounced psychological distress, and a lower score suggests psychological well-being.

The GHQ-12 has undergone translations in 38 languages, including an Urdu version assessed by Riaz & Reza [43] for Pakistan. Based on its validity and effectiveness, GHQ-12 has been adopted as a major tool to assess psychological distress.

### 4.3. Procedure for Data Collection

The research commenced with the initiation of contact with the Rescue headquarters located throughout various districts in Punjab, Pakistan. The primary aim was to obtain the necessary permissions to interact with potential respondents and to facilitate sample collection. Participant selection was guided by specific criteria established for the study, with emphasis on those who demonstrated both eligibility and willingness to participate.

The choice of Punjab’s Rescue Department as the study’s focus was informed by its diverse responsibilities in responding to a wide array of disasters and emergencies. The rescue workers in this region operate under a system of rotating shifts, changing monthly. Consequently, the selected participants had extensive exposure to various types of emergencies and disasters, encompassing natural calamities, road accidents, bomb blasts, shooting incidents, severe domestic situations resulting in death or injury, and critical medical emergencies. This extensive exposure meant that all participating rescue workers had not only directly witnessed trauma but had also absorbed secondary experiences through discussions with colleagues and informal exchanges within their work environment.

Upon successfully liaising with the authorities, the next step was to brief potential participants about this research’s intent and purpose. All the participants were presented with an informed consent document, highlighting the study’s objectives, and providing an overview of the questionnaire procedure. They were assured that their contributions would remain confidential and be used strictly for academic purposes. Once participants acknowledged their understanding and signed the consent form, they were administered the STSS and GHQ-12 questionnaires. These instruments were specifically chosen to gauge levels of secondary traumatic stress and psychological distress, respectively [35,36]. 

The entire data collection phase spanned approximately eight weeks. Each individual interaction was characterized by a face-to-face, one-on-one session lasting around 30 min. Participants were encouraged to answer all questions and were assured that any clarifications or doubts they had would be promptly addressed by the researcher.

Following the data collection, the responses were compiled and processed using the SPSS software (version 28) for in-depth analysis. The specifics of the analysis techniques employed can be found in the results discussion of the study.

### 4.4. Statistical Analysis

To assess the relationships and differences among variables, the study employed a combination of statistical techniques. Pearson correlation analysis was performed to gauge the strength and direction of the linear relationship between secondary traumatic stress (STS) and psychological distress (PD). Given the age segmentation of the participants, *t*-tests were employed to assess any significant differences in STS and PD scores between the two age groups (22–35 years and 35–45 years) [44]. As participants were further divided based on other criteria like exposure to trauma, ANOVA (F-test) was used to analyze differences in STS and PD scores across these categories [45].

## 5. Analysis and Results

### 5.1. Descriptive Statistics of the Sample

Descriptive statistics indicate that a significant proportion of the participants (81.2%) fall within the age bracket of 22–35 years. When examining educational backgrounds, it is evident that a majority (67.5%) have completed an education spanning 9–12 years. Additionally, a vast majority, 91.3%, had considerable work experience—more than 4 years of work experience. In terms of work duration, 61.3% had a longer working hours duration, and a similar trend was observed in training duration to how much training they have obtained during their job, with 81.3% having undergone prolonged training—training of more than 6 months. As for trauma exposure durations, the results were distributed as follows: 45% were exposed for 1–7 days, 26.3% for 8–15 days, 8.8% for 16–30 days, and the remaining 20.0% had experienced trauma for more than 30 days.

### 5.2. Correlation between Secondary Traumatic Stress and Psychological Distress

A Pearson correlation analysis was conducted to determine the relationship between secondary traumatic stress on the secondary traumatic stress scale (STSS), and psychological distress on the general health questionnaire (GHQ-12). The results of this analysis are presented in Table 1. The correlation coefficient, represented as r, was found to be 0.38. The statistical analysis indicates that the correlation, between the two variables is significant with a *p*-value of less than 0.001 (*p* < 0.001). This suggests a significant relationship between them.

The positive correlation found suggests that as scores on the STSS increase, there is an increase in scores on the GHQ-12. This implies that higher levels of stress are associated with higher levels of psychological distress. The analysis indicates that rescue workers who experience traumatic stress are also likely to experience higher levels of psychological distress.

Table 2 presents the results of *t*-tests which are specifically designed to compare means between two groups [44]. In this study the sample has been divided into two groups based on age, work duration, work experience, and duration of training, making *t*-tests an ideal choice. By using *t*-tests, it is possible to ascertain if there is a difference in means between these two groups and obtain a result.

Based on the scores, it seems that rescue workers in the age group of 22–35 years are likely to experience higher levels of secondary traumatic stress (mean score of 35.11) compared to those aged 36–45 years (mean score of 29.07). The *t*-test results indicate a statistically significant difference in STSS scores between the two age groups. Specifically, younger rescue workers (22–35 years) exhibit higher levels of secondary traumatic stress compared to those aged 36–45 years. A similar pattern of findings supported significantly high PD among the younger age group (M = 8.65, SD = 3.289) compared to the older group (M = 1.19, SD = 0.393). However, it is imperative to underscore the potential limitations in the generalizability of these findings, primarily attributable to the disproportionate representation of younger participants within the sample cohort. Consequently, while these results are demonstrative of the specific sample under investigation, they may not necessarily extrapolate to a more balanced demographic distribution encompassing an equitable representation of both younger and older rescue workers. Therefore, this factor needs more research and assessment. 

Rescue workers with longer working hours (10–13 h a day) have appeared to experience higher levels of secondary traumatic stress (mean score of 37.87) compared to those working for fewer hours (mean score of 34.04). Workers having less professional experience (2–5 years) experience more STS (means score 35.25) than workers having more experience (means score 31.14). Similarly, workers who received more duration of training appeared to experience less STS (means score 31.24) than workers having less training duration (means score 35.25).

To find the relationship between work duration and STSS, again Pearson correlation analysis was conducted. The relationship shows positive and significant results with r = 0.21 and *p* < 0.01. This shows that as the work duration increases, STS also increases.

To explore the relationship between “Exposure to Trauma” (quantified by the time of exposure) and the levels of Secondary Traumatic Stress, the sample has been divided into four groups as shown in Table 3 below. As there are more than 2 groups, ANOVA is the optimal choice for this analysis because it is specifically designed to compare the means of three or more groups, making it suitable for the four “time of exposure” groups in this research [44,45]. Rather than using multiple *t*-tests, which increases the risk of Type I errors, ANOVA offers a single, comprehensive test that efficiently examines if there are overall differences among the groups [45]. Additionally, ANOVA is ideal for situations where one independent variable with multiple levels impacts a dependent variable, allowing for a streamlined analysis of the effect of trauma exposure duration on STSS scores.

From Table 3, it can be assessed that the mean STSS scores tend to decrease as the duration of exposure to trauma increases. In simpler terms, the longer a rescue worker is exposed to trauma, the lower their STSS score appears to be, based on the group averages. 

The F-score (or F-statistic) represents the ratio of the variation between the group means to the variation within the groups. A higher F-score indicates a greater likelihood that there’s a real difference between the groups. The F-score of 2.804 suggests that there’s some difference between the group’s means. Specifically, it indicates that the variance between the group means is 2.804 times greater than the variance within the groups.

The ANOVA results suggest that there is a statistically significant difference in STSS scores between at least two of the exposure groups. Given the pattern in the means, it appears that prolonged exposure to trauma is associated with lower STSS scores, although the exact nature or reason for this relationship requires further exploration. A possible explanation for this pattern, though it needs more research to be confirmed, is the idea of psychological adaptation or getting used to something. This means that over time, people might become more accustomed to traumatic events, which could reduce the intense stress reactions that usually happen when they first experience these events [46]. This idea is in line with theories in psychology that suggest people have a natural ability to adjust to ongoing stressful situations, gradually reducing their emotional and psychological reactions. However, the specific ways in which this happens, and the mental processes involved still need to be studied more and explained in detail.

## 6. Discussion

This section aims to contextualize and interpret the findings derived from the data analysis, drawing connections to prior research, and providing deeper insights into the intricate relationships between our variables. Specifically, in this section, we will explore the association between secondary traumatic stress (STS) and psychological distress (PD) among rescue workers, examining the strength and implications of this relationship in light of existing literature.

The findings presented in Table 1 show a significant correlation between secondary traumatic stress (STS) and psychological distress (PD). This observation supports Hypothesis H1, which posited a positive correlation between STS and PD among rescue workers. Such a connection was anticipated, especially given both constructs emerge from exposure to trauma. The results are in line with the research outcomes of Renshaw et al. [31] and Orrù et al. [32], further confirming the intricate relationship between these variables among rescue workers.

Examining in detail, it is worth noting that both STS and PD are constructs stemming from traumatic exposures though in different capacities, but their interlinked nature is undeniable. This interdependence underscores the imperative of understanding their combined influence, especially within the challenging and high-stakes context of rescue operations. Given the unique aspects and trauma exposures inherent to rescue work, a robust exploration of this association is not just academically significant but also holds potential implications for interventions and support systems tailored to this professional group and may extend to other occupations where employees are vulnerable to extreme stressful situations [47].

### 6.1. The Interplay between Age and Secondary Traumatic Stress in Rescue Work

Age, often intertwined with experience, resilience, and coping mechanisms, offers a multifaceted lens through which to view and interpret these psychological constructs, especially in high-intensity professions like rescue work.

The findings, as outlined in Table 2, indicate a noteworthy trend: older rescue workers tend to report lower levels of secondary traumatic stress (STS) compared to their younger counterparts. This observation aligns with our hypothesis H2, emphasizing variations in STS across different age groups. This pattern mirrors findings from Dworkin et al. [31], who noted elevated STS symptoms among younger staff working with trauma survivors in rape crisis centers.

However, the correlation between age and STS isn’t universally accepted. Some studies, like the one by Măirean et al. [48] which assessed 52 healthcare providers, found no discernible link between age and STS. Similarly, a meta-analysis by Hensel et al. [49] and a study by Ogińska-Bulik et al. [20] indicated only a weak association between these variables. The discrepancies in findings across different studies hint at the complex nature of this relationship, suggesting that it is influenced by a myriad of underlying factors.

In the context of this research, centered on rescue workers in Punjab, Pakistan, these contradictions in the broader literature underscore the importance of detailed exploration. Rescue workers, often at the frontline of traumatic events, face unique challenges and stressors. How age influences their susceptibility to STS and PD, and whether it acts as a buffer or a vulnerability factor, is a question of both academic and practical significance. Given the variations in global research outcomes, this study’s findings could provide a more localized understanding, potentially explaining cultural, organizational, or regional factors that modulate the age-STS/PD relationship.

### 6.2. Work Duration’s Influence on Secondary Traumatic Stress

Data from Table 2 and the results of the correlation between STSS and work duration (r = 0.21; *p* < 0.01) indicate that an extended work duration corresponds to increased levels of STS and PD. Specifically, rescue workers logging in longer hours (10–13 h) reported elevated STS compared to those working shorter shifts (7–9 h), validating hypothesis H3. One potential explanation for this phenomenon is that extended work durations could lead to increased fatigue, which in turn exacerbates feelings of stress. Consequently, the longer an individual is engaged in work, particularly in high-stress environments, the more likely they are to exhibit elevated stress responses [50].

This correlation finds support in Hinderer et al. [25], highlighting the interlinking of work duration with trauma exposure frequency and workload intensity, both crucial determinants of STS. Hinderer et al. [25] observed that increased shift hours elevated STS prevalence. Echoing this, Yoder [26] identified that nurses, especially from high-stress departments such as emergency and oncology, displayed intensified STS symptoms when subjected to heavy workloads without adequate breaks.

### 6.3. Impact of Professional Tenure on Secondary Traumatic Stress

Duration or length of professional experience holds a pivotal position when analyzing the susceptibility of individuals to secondary traumatic stress (STS), especially within professions consistently confronting trauma, like rescue work.

The observations, as presented in Table 2, indicate a noteworthy trend: rescue workers with a long history in the field (having more experience) tend to report reduced STS levels compared to their less-experienced colleagues. This pattern supports hypothesis H4, which postulated that the length of work experience significantly influences the incidence of STS and psychological distress among rescue workers.

This inclination aligns well with the findings of Konistan [22]. The study highlighted that while a significant fraction of professionals engaged with trauma-affected patients demonstrated symptoms indicative of STS, the intensity of these symptoms seemed inversely proportional to their duration in the field. Specifically, those relatively new to the profession were more prone to pronounced STS manifestations. Such heightened susceptibility among novices can be attributed to the initial shock and lack of preparedness in confronting traumatic scenarios. On the other side, seasoned rescue professionals, having experienced numerous traumatic exposures over extended periods, often exhibit slight STS. Their apparent resilience and accumulated coping strategies might mask the underlying stress, making it less overt but not necessarily non-existent [22].

This contrast between beginners and veterans emphasizes the importance of structured training and ongoing support mechanisms to equip individuals, regardless of their professional tenure, to effectively manage the toll of STS.

### 6.4. Effect of Training Duration on Secondary Traumatic Stress

The results, as reflected in Table 2, reveal an inverse relationship between the duration of training and the prevalence of STS and PD among rescue workers. Specifically, a longer training span (7–12 months) was associated with reduced levels of STS and PD compared to shorter training periods (1–6 months), thereby supporting hypothesis H5.

These findings are in line with Halamová et al. [23], highlighting training’s two-pronged effect. While comprehensive training emphasizing psychological resilience can effectively mitigate STS susceptibility, an overemphasis on traumatic narratives and, a lack of coping strategies, might inadvertently intensify STS [22]. Focusing on this, Robinson et al., stress the merit of well-designed training frameworks in aiding rescue workers to navigate the challenges of STS and PD [24].

Moreover, the importance of targeted training extends beyond the individual, advocating for systematic organizational strategies. Armes et al. [27] highlighted the significance of training supervisors to identify and address traumatization, reinforcing the role of social support in negating adverse effects. Additionally, Colombo et al. [51] emphasized that training focused on fostering personal resources and self-care can be transformative, not only in aiding individuals but also in propelling organizations toward nurturing positive work environments.

### 6.5. Trauma Exposure and Its Influence on Secondary Traumatic Stress

An intriguing observation from Table 3 indicates an inverse relationship between trauma exposure duration and STSS scores. Specifically, as rescue workers experience prolonged trauma exposure, their reported STSS and PD scores tend to reduce. This supports hypothesis H6, which posited variability in STS and psychological distress among rescue workers contingent upon their trauma exposure. 

This finding is consistent with Armes et al. [27], underscoring the differential impacts of trauma exposure durations on STS manifestations among individuals.

However, this seemingly counterintuitive pattern—that extended trauma exposure might mitigate STS—stands in contrast with several studies. Adriaenssens et al. [28] underscored that professionals confronting heightened trauma caseloads reported exacerbated STS symptoms. Further reinforcing this perspective, a comprehensive meta-analysis by Cieslak et al. [29] indicated that protracted direct trauma exposure escalates both STS and psychological distress.

Despite these contradictions, the core of hypothesis H6 remains validated: the experience of STS and psychological distress among rescue workers is intricately linked with their trauma exposure, as evidenced in Table 3. 

In the context of rescue workers, it may be the case that repeated exposure to phenomena such as incident management, trauma response, and other high-stress situations could lead to a process of habituation or acclimatization [50]. This theory posits that with continuous exposure, individuals may progressively adapt to these stress-inducing circumstances, thereby potentially diminishing their acute stress responses over time [50]. The findings may not be universally applicable or generalizable to other populations with different exposure levels or coping mechanisms. Therefore, while interpreting these results, one must consider the unique occupational context of rescue workers and the Pakistani cultural context, which may inherently foster a degree of desensitization or adaptation to stressful and traumatic events.

Given the research’s unique context—rescue workers in Punjab, Pakistan—it is imperative to further investigate the dynamics of this relationship. Potential factors like cultural nuances, organizational support, or regional influences might offer clarifying insights. Interestingly, comparisons can be drawn between this phenomenon and findings related to work/professional experience, where seasoned rescue workers exhibited diminished STS compared to their less experienced counterparts.

In synthesizing the results and discussions presented, it becomes apparent that the complex interplay between secondary traumatic stress (STS), psychological distress (PD), and various influencing factors like age, training, work duration, and trauma exposure offers insights, especially for the unique demographic of rescue workers in Punjab, Pakistan. While certain findings, such as the correlation between STS and PD, align well with extant literature, others present intriguing contradictions, compelling us to reconsider traditional frameworks and probe deeper. These discrepancies highlight the necessity of locale-specific research, reminding us that while broader trends provide valuable baselines, the intricacies of individual, cultural, and regional contexts can dramatically shape outcomes. As this research moves forward, it becomes imperative not just to understand these findings but to contextualize them within the unique challenges faced by rescue workers, ensuring that the knowledge gleaned here serves as a cornerstone for future interventions, policy decisions, and research endeavors.

## 7. Limitations of the Study

The study, while providing valuable insights, has certain limitations that warrant consideration. One notable constraint is the geographical focus, as the research was confined to rescue workers from the Rawalpindi region of Punjab, Pakistan. This specific geographical concentration might affect the broader applicability of the findings, as rescue workers from other regions or countries may operate within different cultural, organizational, or socioeconomic contexts. 

Additionally, the cross-sectional design of the research captures only a specific moment in time, limiting the ability to observe changes or trends in the participants’ experiences and perceptions over longer durations. Such a design might not encapsulate the evolving nature of secondary traumatic stress and psychological distress among rescue workers. 

Furthermore, the study’s demographic composition exclusively comprised male participants, neglecting the experiences and potential challenges faced by female rescue workers. This gender exclusivity, combined with a relatively modest sample size of just 80 rescue workers, might limit the depth and diversity of perspectives captured, thereby constraining the study’s comprehensive representation and generalizability.

## 8. Implications for Practice

Addressing the psychological toll of traumatic events requires multifaceted approaches. As emphasized by Hamid and Bhat [15], there’s an urgency for community-based health programs that are culturally and gender-sensitive, catering to varied population segments. Recognizing the noticeable connection between secondary traumatic stress (STS) and psychological distress (PD), organizations should prioritize the mental well-being of their workers. By acknowledging the vital role that age, work duration, and training play in influencing STS, institutions can tailor their induction, training, and support processes. For instance, newer entrants to the field, who this study found may be more vulnerable, can be offered specialized training modules and mentorship programs to enhance their resilience.

Further, the critical role of training duration in moderating STS and PD underscores the need for comprehensive and ongoing training programs. Such initiatives shouldn’t only focus on the technical aspects of the job but also incorporate psychological resilience and coping strategies. It is not just about preparing workers for the physical challenges but also equipping them mentally. Given the nuanced relationship between trauma exposure and STS, institutions should regularly review and if necessary, recalibrate their operational guidelines. This is particularly crucial in terms of workload management, ensuring adequate rest intervals between shifts and, perhaps, introducing rotational job profiles to minimize consistent direct exposure to trauma.

Moreover, the gender exclusivity observed in this study highlights the need to diversify and explore the experiences of all gender groups in the rescue workforce. Incorporating diverse perspectives can lead to a more holistic understanding and formulation of support mechanisms. In essence, while the results provide a foundation, they also call for a more holistic, inclusive, and dynamic approach to organizational practices concerning rescue workers.

## 9. Conclusions and Future Directions

Drawing from the insights of this study, it is evident that the mental well-being of rescue workers is intricately tied to various factors, including age, duration of professional exposure, training, and direct interaction with traumatic events. Secondary traumatic stress (STS) and psychological distress (PD) emerge not just as standalone issues, but as interconnected phenomena influenced by the above factors. The importance of these findings cannot be overstated in the context of rescue work, where the very essence of the job demands closeness to traumatic events. The influence of training, both its quality and duration, has been especially highlighted, underscoring the pivotal role it plays in shaping the psychological resilience of rescue workers.

While this study illuminated several aspects, the gender-limited sample demands a broader study encompassing all gender groups to fully comprehend the landscape of STS and PD among rescue workers. Moreover, while this research shed light on the association of various factors with STS and PD, longitudinal studies could provide insights into the causality and the progression of these psychological states over time.

Furthermore, future studies should also delve into the efficacy of various intervention strategies in mitigating STS and PD. Identifying, validating, and implementing these strategies will play a crucial role in ensuring the psychological well-being of those who stand at the forefront during crises.

## Figures and Tables

**Table 1:** Pearson correlation between secondary traumatic stress (STS) and GHQ-12 (N = 80).

**Secondary Traumatic Stress** **STSS**	**Mean (STSS)**	**SD (STSS)**	**Mean (GHQ)**	**SD (GHQ)**	**GHQ Correlation**	
					**r**	***p***
	33.62125	7.45075	9.52	6.18	0.38	0.00

**Table 2:** STSS Mean (M), Standard Deviation (SD), and *t*-value of age, time of exposure/work duration, work experience, and duration of training of groups of participants.

Variable	Group	Mean (M)	Standard Deviation (SD)	*t*-Value	*p*
Age	22–35 Years	35.11	7.661	2.72	0.01
	36–45 Years	29.07	8.031		
Work Duration	7–9 h	34.04	8.175	2.15	0.03
	10–13 h	37.87	7.731		
Work Experience	2–5 Years	35.25	8.353	2.52	0.01
	6–10 Years	31.14	6.151		
Duration of Training	1–6 Months	35.25	7.353	2.6870	0.00
	7–12 Months	31.24	6.151		

**Table 3:** Mean differences between secondary traumatic stress (STSS) and time of exposure from trauma (N = 80).

Variable	Group	Mean (M)	Standard Deviation (SD)	*F*	*p*
Time of Exposure	1–7 Days	35.44	7.485	2.804	0.02
	8–15 Days	34.76	8.831		
	16–30 Days	31.01	6.164		
	More than 30 Days	28.56	9.251		

## Data Availability

The data presented in this study are not publicly available to maintain the confidentiality and privacy of the people who participated in the study.
